# A Benign Finding of Hemorrhagic Pancreatitis

**DOI:** 10.7759/cureus.70339

**Published:** 2024-09-27

**Authors:** Andrej M Sodoma, Eric Stone, Andrea Schmitt, James R Pellegrini, Jaspreet Singh

**Affiliations:** 1 Internal Medicine, South Shore University Hospital, Bay Shore, USA; 2 Internal Medicine, Rocky Vista University College of Osteopathic Medicine, Englewood, USA; 3 Internal Medicine, Nassau University Medical Center, East Meadow, USA; 4 Gastroenterology, South Shore University Hospital, Bay Shore, USA

**Keywords:** critically ill patients, hemorrhagic shock, pancreatic mass, severe pancreatitis, surgical emergency

## Abstract

Hemorrhagic pancreatitis is a rare cause of hypovolemic shock. It presents as silent bleeding, with signs of hypovolemic shock and abdominal pain eventually culminating in life-threatening bleeding. This case study delves into a case of hemorrhagic pancreatitis in a 49-year-old male. Notably, he has a history of recurrent lower extremity (LE) deep vein thrombosis (DVT) and atrial fibrillation (AF) on Coumadin. He came in with shortness of breath (SOB) and was admitted for acute hypoxic respiratory failure secondary to Influenza A. A few days into admission, the patient developed acute cardiogenic shock, septic shock, and acute respiratory distress syndrome (ARDS). The patient developed rectal bleeding with a decrease in hemoglobin and hematocrit. A computed tomography (CT) of the chest, abdomen, and pelvis (CAP) without (w/o) contrast was performed to find a source. It showed hemorrhagic pancreatitis in the head/tail region. The bleeding resolved on its own without interventions or blood transfusion. Hemorrhagic pancreatitis carries a high mortality rate. In this case, it had an insidious onset with self-resolution, a rare case. Physicians should make quick referrals for surgical resection in hopes of better outcomes.

## Introduction

We present a case in which a middle-aged male had self-resolving hemorrhagic pancreatitis. Hemorrhagic pancreatitis is a late sequela of acute pancreatitis where necrotizing pancreatitis or pancreatic pseudoaneurysm rupture results in bleeding, most often into the retroperitoneum or peritoneal cavity [1]. Necrosis and enzyme leakage can cause pseudoaneurysm formation as pancreatic enzymes break down vessel walls, often of the splenic, gastroduodenal, and pancreaticoduodenal arteries [2,3]. This condition carries high mortality in large part due to massive hemorrhage. Mortality of hemorrhagic pancreatitis is anywhere from 20% to >60% [4,5,6]. Clinical features suspect the diagnosis, principally the presence of the Cullen sign or Grey Turner sign, particularly with historical features like epigastric pain, vomiting, and hemorrhagic shock [7]. Confirmation is performed with magnetic resonance imaging (MRI), which may be more accurate than CT [8]. Workup should demonstrate elevated serum lipase. Still, there are reported cases of acute pancreatitis with normal serum lipase [9]. This patient is unique in that hemorrhagic pancreatitis was identified on imaging and resolved without surgical intervention, which is rare for hemorrhagic pancreatitis as it typically has a high mortality rate and requires emergent surgical intervention. 

## Case presentation

A 49-year-old man from Eastern Europe presented to the emergency department (ED) with shortness of breath and fever. While in the ED, the patient had a non-productive cough, shortness of breath on exertion, slight hypoxia of SpO_2_ of 91% requiring a 2-L nasal cannula, no leukocytosis or anemia, and electrolytes within normal limits. The only positive result was a nasal swab respiratory viral panel, which showed Influenza A H1 2009. The patient then left the ED against medical advice the following morning and returned later that day due to continued shortness of breath. In the ED, he had escalating oxygen requirements with tachypnea and increased work of breathing, requiring bilevel positive airway pressure (BiPAP) 12/6 at 60% fraction of inspired oxygen (FiO_2_). He was admitted to the medical intensive care unit (MICU) for the management of worsening acute hypoxic respiratory failure.

While in the MICU, the patient had a prolonged and complicated stay. The patient was persistently in AF RVR refractory to was amiodarone load and drip (150 mg intravenous [IV] was given once, followed by 1 mg/minute IV Amiodarone continuous infusion) and direct current cardioversion (DCCV). The patient was on heparin full AC for AF. The patient had a persistent shock state requiring two vasopressors (norepinephrine bitartrate and vasopressin). The patient was in biventricular heart failure (HF) with an ejection fraction (EF) of <20%, moderate right and left atrial dilation, moderate pulmonary hypertension (pHTN) of 51 mmHg, and mild tricuspid and mitral regurgitation. The persistent shock state caused renal failure and ischemic hepatitis. The patient was progressively weaned off pressors as tolerated. Urine output improved, so the patient transitioned to intermittent hemodialysis. The patient still required a ventilator, so a tracheostomy was placed. After a few days, the shock liver resolved, allowing for amiodarone load, improving cardiac output, and metoprolol soon after since the patient was no longer on vasopressors.

Midway through his third week of hospitalization, the patient developed rectal bleeding with mild, dull, epigastric abdominal pain. Hemoglobin/hematocrit (H/H) was low at Hgb 9.7 g/dL (normal range: 13.5-17.5 g/dL) and Hct 30.5 (normal range: 40%-54%) but not worsening and was not an acute drop. The patient was 13.6/42.6 five days before the trough; on the fourth day before the trough, the H/H was 11.9/37.2. On the third day, it was 11.4/36.4; on the second day, it was 10.8/34.3; and on the day before, it was 10.4/32.6; during this time, the patient had no rectal bleeding or abdominal pain until the morning of the value of the trough. Also, due to the biventricular HF, persistent AF, and prolonged MICU stay, it was difficult to see any signs of symptomatic anemia as the patient was persistently tachycardic, hypotensive, and lethargic.

**Figure 1:**
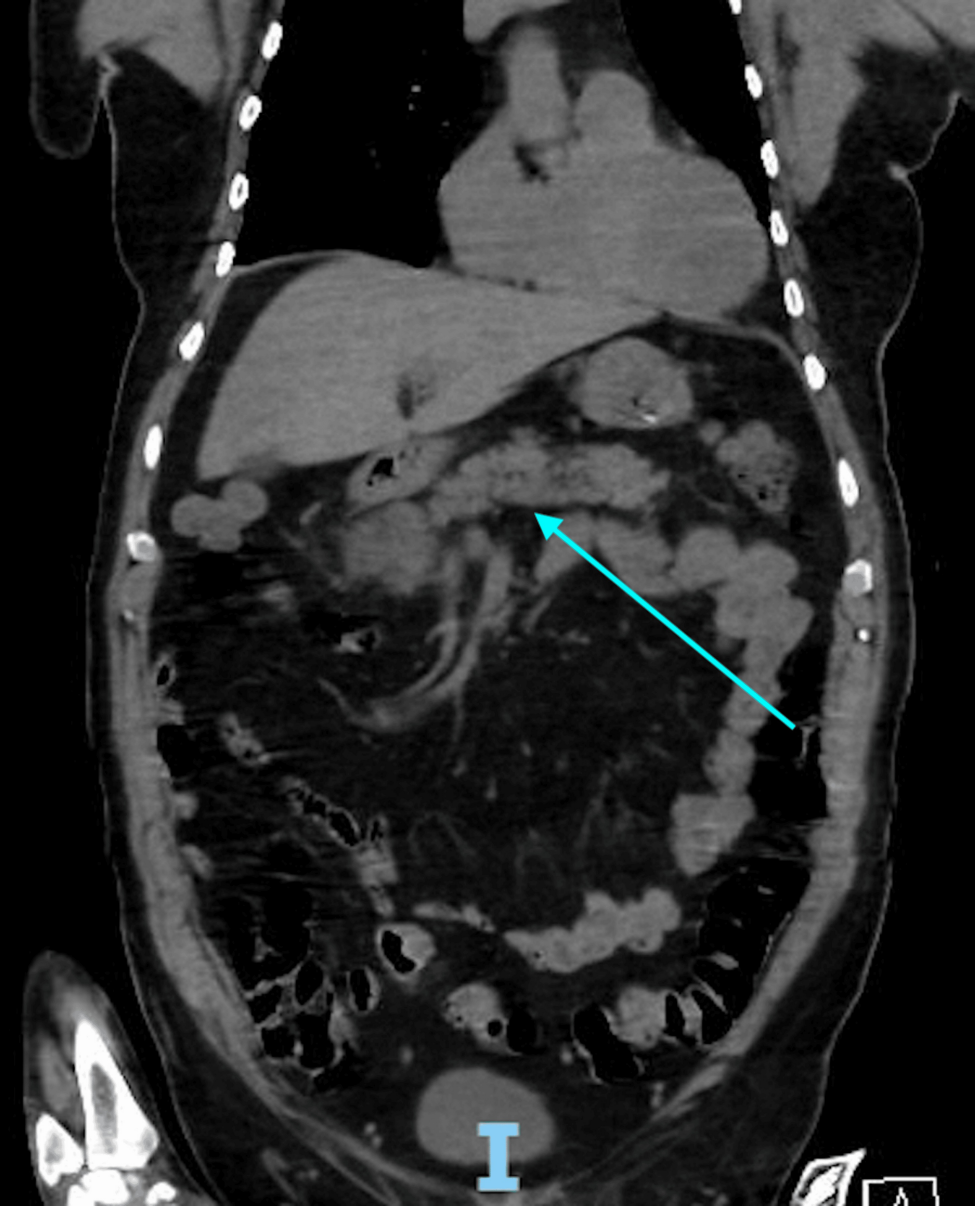
Computer tomography of the chest, abdomen, and pelvis without contrast, in the coronal plane view. The arrow pointing toward findings of pancreatitis, including high attenuation peripancreatic fluid collections reflecting hemorrhagic pancreatitis.

**Figure 2:**
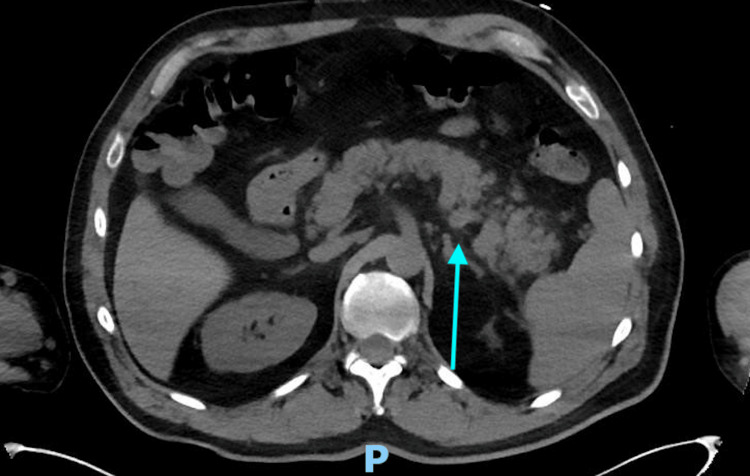
Computer tomography of the chest, abdomen, and pelvis without contrast transverse view. The transverse view of the image showing signs of pancreatitis with signs of hemorrhagic pancreatitis.

The patient underwent CT of the chest/abdomen/pelvis (C/A/P), which revealed fluid collection containing high attenuation material adjacent to the pancreatic head and tail, with the most considerable portion 5 cm in size at the tail. It was determined to be hemorrhagic pancreatitis (Figures 1-2). A lipase was measured once the CT was read. Lipase was 191 U/L (normal range: 0-160 U/L) at the initial measurement, 161 U/L three days later, and 82 U/L one week after the initial measurement. The mass was monitored. Gastroenterology was consulted when the finding was found, and no interventions were performed as the mass did not change in size; the patient remained stable, had no abdominal pain, and had no further signs of anemia. The patient HF was worked up with a left heart catheterization, which showed non-obstructive CAD with reduced EF of <20%. The patient was gradually placed on GDMT as tolerated. Tracheostomy was decannulated six weeks after admission. The patient was discharged to subacute rehab (SAR) after seven weeks in the hospital. After two weeks in SAR, the patient was discharged home and followed up with cardiology afterward. The patient had no abdominal pain. The patient complained of fatigue but is improving and is no longer in Afib RVR. Heart failure reduced ejection fraction (HFrEF) has not improved, but is complying well with medications.

## Discussion

Pancreatic necrosis and enzyme leakage can lead to the formation of pseudoaneurysms due to the destructive effects of pancreatic enzymes on vessel walls. This condition carries a high mortality rate, necessitating prompt evaluation and management. Patients with suspected hemorrhagic pancreatitis should be worked up similar to that of acute pancreatitis (AP), with additional imaging (CT or MRI) to detect bleeding. The patient, in this case, had an incidental finding of hemorrhagic pancreatitis. The etiologies of hemorrhagic pancreatitis parallel those of acute pancreatitis (AP), including chronic alcohol abuse, gallstones, infection, and hypertriglyceridemia, with additional causes such as chronic pancreatitis, trauma, biliopancreatic surgery, and pancreatic transplantation. Diagnostic criteria for pancreatitis typically involve elevated serum lipase levels, characteristic abdominal pain, and imaging findings. Imaging remains crucial in cases with high clinical suspicion but normal enzyme levels, which may indicate a delayed presentation [10]. In this case, a lipase of 191 U/L was obtained when CT demonstrated HP and the patient had epigastric pain. This value is lower than the greater than 480 U/L value used as criteria for AP. A subsequent decrease to 82 U/L within a week suggested improved condition. A cause in this case is multifactorial, resulting from pneumonia, persistent shock state, and prolonged AC use. Hemorrhagic pancreatitis can cause uncontrolled retroperitoneal or peritoneal bleeding, which is often fatal. Limited research complicates the establishment of definitive treatment protocols. Early referral for surgical evaluation is typically necessary [11,12]. In this case, it was an incidental finding and spontaneously resolved, making surgical involvement unnecessary. 

Imaging is critical in diagnosing hemorrhagic pancreatitis and determining the need for surgical intervention. Early assessment with contrast-enhanced multidetector computed tomography (MDCT) or MRI is recommended [10,13]. Hemorrhage can also be aged qualitatively by assessing the attenuation value as the hematoma ages [13]. Additionally, urinary trypsinogen activation peptide (TAP) and urinary trypsinogen-2 may provide information regarding the severity of pancreatitis and the risk of hemorrhagic complications [14,15]. 

Outcomes improve with early detection and prompt evaluation for angiography, embolization, or surgery associated with decreased mortality [2,6]. Addressing pseudoaneurysms, when present, may also enhance survival. Endovascular interventions, such as coil embolization and liquid embolic agents, have effectively treated these defects [11,12] The benefit of prophylactic antibiotic use in this condition is unclear. Given the association with AP, managing this underlying condition may decrease the risk of bleeding. Some studies report improvement in severe AP with imipenem, imipenem/cilastatin, or ciprofloxacin/metronidazole (Flagyl) [16]. However, other research demonstrates no benefit with ciprofloxacin/metronidazole (Flagyl), meropenem, beta-lactams, or quinolones with imidazole [17]. One review proposed an algorithm for managing hemorrhagic pancreatitis based on the location and severity of bleeding [18]. Early surgical consideration is critical in the management of hemorrhagic pancreatitis. 

## Conclusions

Hemorrhagic pancreatitis is a rare cause of hypovolemic shock that is a surgical emergency with a high rate of mortality. This case highlights a rare instance of an incidental finding of hemorrhagic pancreatitis with slight anemia. Also, with spontaneous resolution of hemorrhagic pancreatitis in a critically ill patient. This offers valuable insights into the clinical identification and management of hemorrhagic pancreatitis.
